# CT and MRI Aspects of an Abdominal Hemophilic Pseudotumor

**DOI:** 10.5334/jbr-btr.887

**Published:** 2015-12-30

**Authors:** M. V. Dupont, E. E. Coche

**Affiliations:** 1 Université catholique de Louvain, CHU UCL Namur, Département de medicine aiguë et medico-technique, Service de radiologie, Avenue Dr G. Thérasse 1, B5530 Yvoir, Belgium; 2 Université catholique de Louvain, Cliniques universitaires Saint-Luc, Department of Radiology, Brussels, Belgium

**Keywords:** Hemophilia, hemophilic pseudotumor

## Abstract

We report the computed tomography (CT) and magnetic resonance imaging (MRI) aspects of a rare case of a patient with a large abdominal hemophilic pseudotumor, a chronic, encapsulated, slowly expanding hematoma occurring in severe hemophilia, without involvement of iliopsoas muscles and iliac bones.

## Introduction

Hemophilic pseudotumor (HP) is a known complication of severe hemophilia (percentage of normal factor activity in blood of less than 1%) [1]. It is a chronic, encapsulated, slowly expanding hematoma occurring in the soft tissues and/or bone. The vast majority of reported cases in the international literature describe musculoskeletal involvement. However, some cases have been reported in the abdomen [2,3,4]. In this unusual location, all of the published clinical cases were described as involving the iliac bones or iliopsoas muscles.

Very few cases of intraabdominal HP without any muscular or skeletal involvement have been reported so far [5], with limited imaging of the pathology. We report the CT and MRI aspects of a patient with a large abdominal HP.

## Case Report

A 65-year-old man suffering from severe and sporadic hemophilia A developed a large abdominal mass 15 years before seeking treatment.

The patient’s medical history included among other noteworthy facts transfusion-related chronic hepatitis C, multiple hemophilic arthropathies, and cerebral hemorrhage.

Laboratory findings were as follows: partial thromboplastin time, 81 sec (normal values 25 to 39 sec), and factor VIII assay, 1%, without inhibitors (normal values 50% to 200%).

The treatment of his hemophilia consisted of two injections of 2000 units of plasmatic factor VIII per week. His hepatitis C was left untreated.

He was referred to our Department of Medical Imaging for the characterization of the mass. Abdominal computed tomography (CT) and a magnetic resonance imaging (MRI) were performed to document this abdominal lump. No ultrasound was performed.

## Imaging Findings

**Figure 1:**
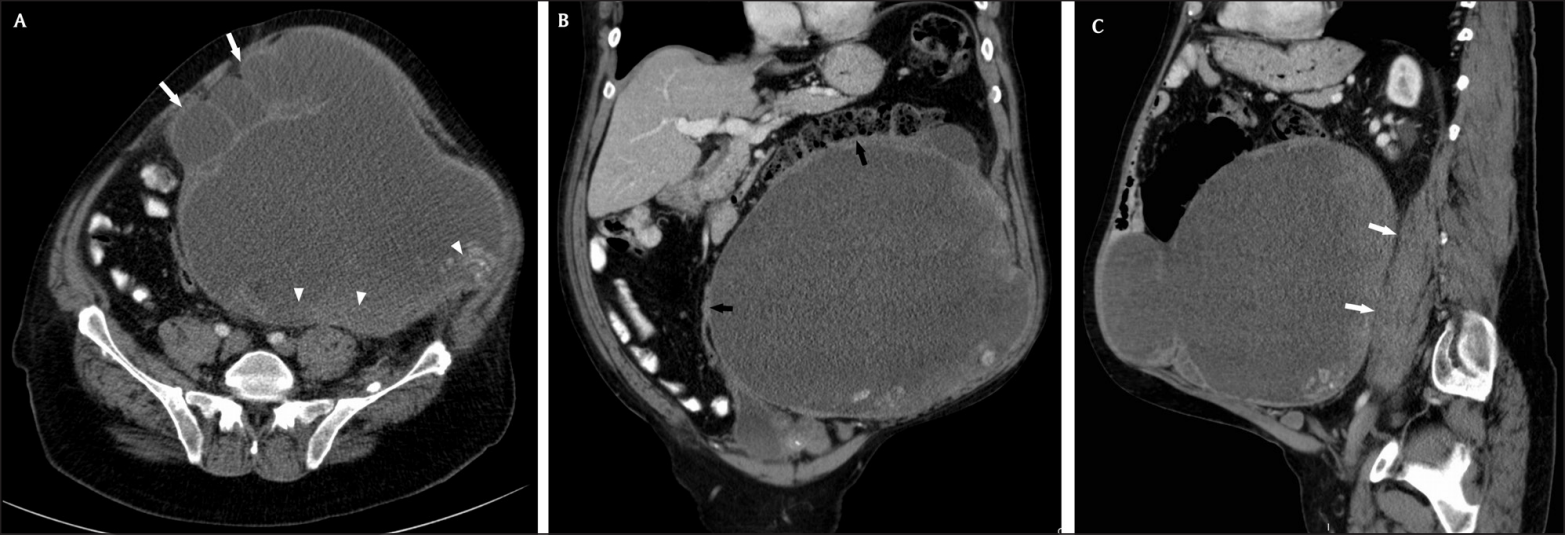
Contrast-enhanced abdominal CT. A. Axial view showing a large encapsulated abdominal mass of low density surrounded by a thin capsule. Lobulated contours and satellite lesions are present at the anterior portion of the mass (white arrows). A dense material is visible at its dorsal part (arrowheads). B. Coronal view, showing the displacement of large and small bowel upward and to the right (black arrows). C. Sagittal view, showing the persistence of fat between the mass and the psoas muscle (white arrows).

CT revealed a large, well-defined, hypodense (10 HU) tumoral process (22 × 22 × 25 cm) with dense gravity-dependent material and calcifications (Figure 1a). This mass occupied the left flank, shifting the left kidney upward, displacing the large and small bowel to the right, and meeting the bladder and the abdominal wall muscles. There was no contact between the mass and left psoas muscle and adjacent bones (Figure 1c).

**Figure 2:**
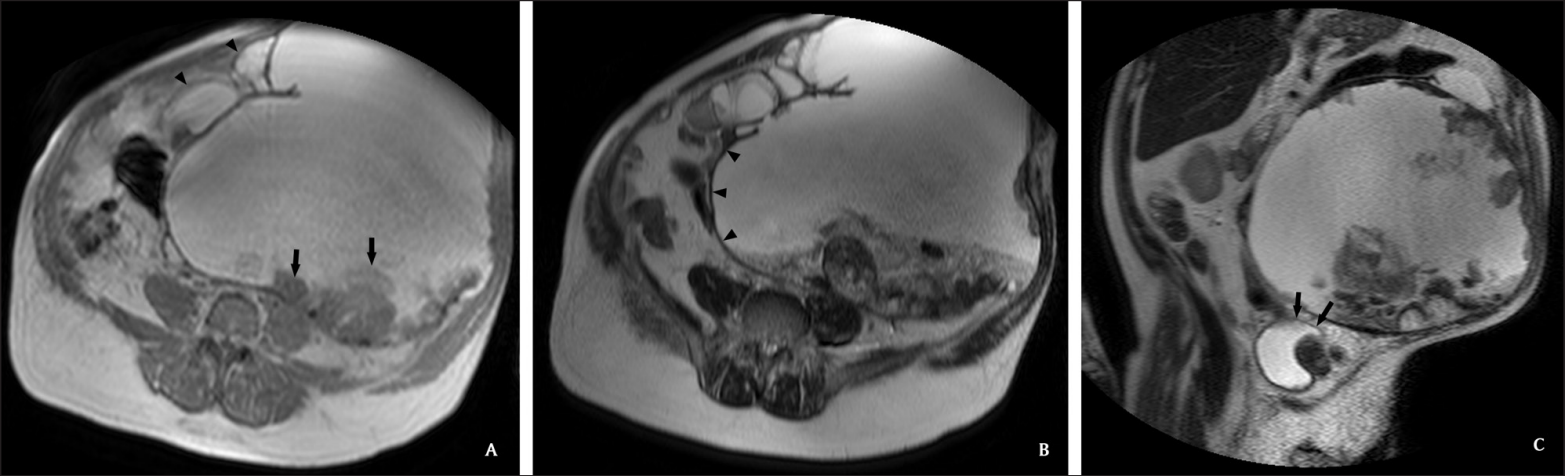
Abdominal MRI. A. Axial T1-weighted gradient echo [175 ms/4.6 ms/60° (TR/TE/Flip angle)], showing the mass with lobulated contours and satellite lesions (black arrowheads), and hyperintense content with posterior more hypointense material (black arrows). B. Axial T2-weighted TSE [8000 ms/80 ms (TR/TE)], showing hyperintense content of the mass with heterogeneous posterior material. The capsule of the mass was more clearly depicted on this sequence (black arrowheads). C. Coronal T2-weighted TSE image, showing the contact between the mass and the bladder (black arrows).

On MRI, the abnormal process showed high T1- and T2-weighted signal intensity, with a less intense posterior material. A capsule of low T1 and T2 signal, more clearly visible on T2-weighted images, surrounded the whole mass, with some satellite nodules, mainly in the lower part of the tumor, near the bladder (Figure 2c).

The diagnosis of HP was suspected because of the context and imaging findings. However, other retroperitoneal tumors, such as mucinous cystic tumors, liposarcoma, or necrotic leiomyosarcoma [6] could not be excluded. Even if biopsy is usually contraindicated because of the potential complications (hemorrhage, infection, and fistula), a biopsy of the mass was performed, revealing the presence of red blood cells with hemosiderin and a fibrous capsule, consistent with the diagnosis of HP.

The follow-up of the patient, through CT and MRI, showed only a slight enlargement of the mass over 3 years.

## Discussion

HP is a complication of severe hemophilia A and B, and type-3 von Willebrand diseases. It is defined as a slowly growing, well-defined mass containing blood clots in various stages of evolution, surrounded by a fibrous capsule [1].

The siege of HP can be intraosseous, subperiosteal, or soft tissue [7]. Soft-tissue pseudotumors can be further classified as intramuscular or extramuscular [8].

On CT, the appearance of HP is that of a mass of low density (10–35 HU), sometimes containing coarse calcifications, and surrounded by a fibrous capsule [2].

On MR, soft-tissue HP appear as masses of variable T1 and T2 signal intensities, corresponding to blood in various stages of evolution, surrounded by a capsule of low T1 and T2 signal intensity, more clearly seen on T2-weighted imaging [8]. Mural nodules are commonly found in soft-tissue pseudotumors [8].

The presence of fat, myxoid stroma (tissue of high T2 signal intensity showing delayed enhancement) or extensive necrosis into the lesion should rather orient the diagnosis to well-differentiated liposarcoma, myxoid tumor, and necrotic leiomyosarcoma, respectively [6].

Complications of abdominal HP include compression of vessels, nerves, and ureters; colonic obstruction; infection; and fistula to skin or large bowel [3,4].

There is no wide agreement among authors on how to manage an HP. Fine needle aspiration is usually contraindicated because of the risk of fistula formation and infection. Surgery or conservative treatment must be evaluated on a case-by-case approach [1,9].

## Conclusion

In patients with known severe hemophilia or von Willebrand disease, the presence of a lesion consisting of blood at various stages of decomposition surrounded by a fibrous capsule should evoke the diagnosis of HP and should not warrant a biopsy regarding the potential complications.

## Competing Interests

The authors declare that they have no competing interests.
